# Genome Sequence for Candida albicans Clinical Oral Isolate 529L

**DOI:** 10.1128/MRA.00554-19

**Published:** 2019-06-20

**Authors:** Christina A. Cuomo, Saranna Fanning, Sharvari Gujja, Qiandong Zeng, Julian R. Naglik, Scott G. Filler, Aaron P. Mitchell

**Affiliations:** a Broad Institute of MIT and Harvard, Cambridge, Massachusetts, USA; b Department of Biological Sciences, Carnegie Mellon University, Pittsburgh, Pennsylvania, USA; c Centre for Host-Microbiome Interactions, Faculty of Dentistry, Oral & Craniofacial Sciences, King's College London, London, United Kingdom; d Division of Infectious Diseases, Los Angeles Biomedical Research Institute at Harbor-UCLA Medical Center, Torrance, California, USA; Vanderbilt University

## Abstract

The diploid heterozygous yeast Candida albicans is the most common cause of fungal infection. Here, we report the genome sequence assembly of the clinical oral isolate 529L. As this isolate grows as a commensal, this genome will serve as a reference for experimental and genetic studies of mucosal colonization.

## ANNOUNCEMENT

Candida albicans is a major cause of bloodstream infections in immunocompromised individuals but is more commonly found in the human mycoflora. Here, we sequenced and assembled the genome of the 529L isolate, which was collected from a patient with oral candidiasis at Guy’s Hospital, United Kingdom (1). In a model of persistent oral and vaginal colonization, the 529L isolate colonized these sites for over 5 weeks with higher fungal burden than with other clinical strains, including SC5314 (1). The 529L isolate has been used in mouse models of oral and vaginal colonization (1–4).

Cells were grown overnight at 30°C in four 4-ml cultures of YPD broth (1% [wt/vol] Difco yeast extract, 2% [wt/vol] Bacto peptone, 2% [wt/vol] dextrose) with shaking. DNA was prepared with the Qiagen Genomic-tip 100/G (catalog number 10243) using the Qiagen genomic buffer set (catalog number 19060), following the manufacturer’s yeast protocol. Cell wall digestion was accomplished with lyticase (catalog number L2524; Sigma). DNA preparations from the four cultures were pooled.

For genome sequencing, two libraries were constructed from genomic DNA. For an ∼180-base-insert library, 100 ng of genomic DNA was sheared to a median size of ∼250 bp using a Covaris LE instrument; the resulting fragments were cleaned using SPRI AMPure XP beads, followed by end repair, A-base addition, and adapter ligation (New England BioLabs) (5). A ∼3-kb insert library was prepared using the 2- to-5-kb-insert mate pair library prep kit (V2; Illumina). Libraries were sequenced on the Illumina HiSeq 2000 platform to generate paired 101-base reads totaling 35,388,222 reads of average quality 34.4 for the 180-base library and 52,230,472 reads of average quality 36.7 for the 3-kb library. Based on an evaluation of assemblies of C. albicans genomes at different coverage levels (6), a subset of approximately 100× sequence coverage of both libraries (28,316,832 total reads) was error corrected, filtered, and assembled using ALLPATHS (7) version R45597 with parameters HAPLOIDIFY=True and ASSISTED_PATCHING = 2.1. The quality of the assembly was evaluated using GAEMR v0.1.0 (https://github.com/broadinstitute/GAEMR); sequencing coverage appeared to be even across the assembly, with no large regions of aneuploidy noted. Three contigs that GAEMR identified as having sequence similarity to mitochondrial sequences were removed from the assembly. The final assembly with 176× read depth includes 87 scaffolds consisting of 608 contigs, with a scaffold *N*_50_ value of 1.2 Mb and a contig *N*_50_ value of 65.5 kb. The total scaffold length is 14.7 Mb, and the average GC content is 33.5%; this is similar in size and GC content to other C. albicans genomes (6, 8).

The genome was annotated by transferring gene coordinates from the SC5314 reference version A21-s02-m01-r01 curated by the *Candida* Genome Database (9) using unique NUCmer (MUMmer v3 [10]) alignments. In cases where the alignment-based mapping resulted in a gene with internal frameshifts, a transcript predicted by Prodigal v2.5 (11) or the longest overlapping open reading frame was substituted if it was longer than the mapped gene. For reference genes not mapped by this method, BLAST v2.2.25 alignments were used to identify missing loci, and gene structures were added using Prodigal v2.5 and GeneWise v2.2.0 (12). A total of 6,211 protein-coding genes were predicted using this approach, similar to the gene content of other C. albicans genomes (6, 8).

### Data availability.

The C. albicans 529L assembly and annotation reported here are available in GenBank under accession number ASHC00000000. Raw sequence reads have been deposited in the NCBI Sequence Read Archive under accession numbers SRX276261 and SRX276262.
